# Breast cancer treatment and survival differences in women in remote and socioeconomically disadvantaged areas, as demonstrated by linked data from New South Wales (NSW), Australia

**DOI:** 10.1007/s10549-021-06170-2

**Published:** 2021-03-21

**Authors:** Elizabeth Buckley, Elisabeth Elder, Sarah McGill, Zahra Shahabi Kargar, Ming Li, David Roder, David Currow

**Affiliations:** 1 Cancer Epidemiology and Population Health Research Group, Allied Health & Human Performance, University of South Australia, Adelaide, Australia; 2 Specialist Breast Surgery, Westmead Breast Cancer Institute, Westmead, NSW 2145 Australia; 3 Cancer Institute NSW, Level 4, 1 Reserve Road, St Leonards, NSW 2065 Australia

**Keywords:** Breast cancer survival, New South Wales, Breast cancer treatment, Socioeconomic status, Residential remoteness

## Abstract

**Introduction:**

Reducing variations in cancer treatment and survival is a key aim of the NSW Cancer Plan. Variations in breast cancer treatment and survival in NSW by remoteness and socioeconomic status of residence were investigated to determine benchmarks. Reducing variations in cancer treatment and survival is a key aim of the NSW Cancer Plan. Variations in breast cancer treatment and survival in NSW by remoteness and socioeconomic status of residence were investigated to determine benchmarks.

**Methods:**

A retrospective cohort study used linked data for invasive breast cancers, diagnosed in May 2002 to December 2015 from the NSW Cancer Registry, with corresponding inpatient, and medical and pharmaceutical insurance data. Associations between treatment modalities, area socioeconomic status and residential remoteness were explored using logistic regression. Predictors of breast cancer survival were investigated using Kaplan–Meier product-limit estimates and multivariate competing risk regression.

**Results:**

Results indicated a high 5-year disease-specific survival in NSW of 90%. Crude survival was equivalent by residential remoteness and marginally lower in lower socioeconomic areas. Competing risk regression showed equivalent outcomes by area socioeconomic status, except for the least disadvantaged quintile, which showed a higher survival. Higher sub-hazard ratios for death occurred for women with breast cancer aged 70 + years, and more advanced stage. Adjusted analyses indicated more advanced stage in lower socioeconomic areas, with less breast reconstruction and radiotherapy, and marginally less hormone therapy for women from these areas. Conversely, among these women who had breast conserving surgery, there was higher use of chemotherapy. Remoteness of residence was associated in adjusted analyses with less radiotherapy and less immediate breast reconstruction. In these short term data, remoteness of residence was not associated with lower survival.

**Conclusion:**

This study provides benchmarks for monitoring future variations in treatment and survival.

## Introduction

Breast cancer is the leading cancer reported in women by Australian cancer registries and second to lung cancer as the leading cause of cancer death in women [1]. Substantial increases in survival from breast cancer in Australia have been recorded, largely attributed to advances in systemic therapies and population-based screening [2]. However, as for many chronic diseases, survival estimates vary across the population [2].

Increased socioeconomic disadvantage and geographical remoteness have been associated with poorer health in many countries, including in Australia [3]. Australia has many geographically remote areas where there may be barriers to accessing health services. Residents living remotely have been found to be diagnosed with breast cancer at a later stage than those in major cities [4]. Also, women living in areas of socioeconomic disadvantage have been less likely to participate in screening and more likely to experience poorer outcomes [4–7].

A principal aim of the NSW Cancer Plan is to improve survival and reduce variation across the NSW population [8]. A range of interventions is used, including provision of ongoing feedback to services from health-service monitoring with linked data [9]. Feedback indicates differences in clinical practice and outcomes that warrant further exploration. Evaluation studies are undertaken regularly to quantify trends in variations and inform planning cycles.

This study is one such evaluation. It investigates invasive breast cancer treatment and survival across areas of differing socioeconomic status and residential remoteness using linked population-based cancer registry notifications. Treatment categories studied included: treatment by surgery; treatment by surgery type (breast conserving and mastectomy); adjuvant radiotherapy following breast conserving surgery (a guideline recommendation); use of adjuvant chemotherapy or hormone therapy where clinically indicated; and reconstruction following mastectomy.

## Methods

Data on histologically confirmed breast cancers (ICD-O-3 C50) from the NSW Cancer Registry (NSWCR) were linked to the NSW Admitted Patient Data Collection (APDC), health insurance claims data from the Medicare Benefits (MBS) and Pharmaceutical Benefits (PBS) schemes, and data from the National Death Index to include deaths occurring outside NSW, as described previously [10, 11].

Notification to the NSWCR of new cancers affecting NSW residents is mandatory under the Public Health Act [12]. Sources of notifications include pathology laboratories, public and private hospitals, imaging centres, aged care facilities and official death registrations from the NSW Registry for Births, Deaths and Marriages [13]. In NSW and other Australian States and Territories, the underlying cause of death is determined by the medical practitioner certifying the death[14].

Linked APDC records include women’s demographic descriptors and information on cancer, other diagnoses and in-hospital procedures performed in NSW public and private hospitals. Principal and additional procedures were identified from Australian Classification of Health Interventions procedural codes [14]. Linked medical claims data were obtained for medical services and procedures, and publicly funded dispensed medications, respectively [15, 16].

For this study, female breast cancers diagnosed from 1st May 2002 until 31st December 2015 were included. Some were excluded where breast cancer surgery (breast conserving or mastectomy) preceded cancer diagnosis by more than 30 days. Also, women from five local health districts adjacent to the NSW border (12%) were excluded for the analysis of treatment only, because NSW information systems did not cover treatment provided outside NSW.

Data were linked as described in the NSW Cancer Plan [8]. Linkage was performed by the Centre for Health Record Linkage for NSW-based datasets and by the Australian Institute of Health and Welfare for linkage to Commonwealth-based datasets [11]. Data were stored in the SURE facility, a remote access computing environment to which authorized analysts were given encrypted access with strong authentication [17].

Demographic data were classified by age at diagnosis (< 40, 40–49, 50–59, 60–69, 70–79, 80 + years) and country of birth (Australia, other mainly English speaking and mainly non-English speaking countries)[10]. Additionally, the Index of Relative Socioeconomic Disadvantage (IRSD) and Accessibility/Remoteness Index of Australia (ARIA) were used as area-level indices of socioeconomic disadvantage and remoteness of residence, respectively [18, 19].

First surgery after diagnosis was identified and classified as whether performed within 12 months following diagnosis (or in the 30 days prior to diagnosis to allow for delays in notification), using APDC procedural codes from the Australian Classification of Health Interventions (ACHI 8th edition) [20]. Breast surgeries identified from MBS item numbers, were classified as breast conserving surgery or re-excision, and mastectomy, and used to supplement APDC records [15]. Breast surgery performed within 12 months of diagnosis was classified as breast conserving (including re-excisions) or mastectomy. Women were also classified as having or not having breast surgery within this period.

Radiotherapy was identified from APDC data, plus radiotherapy centre and MBS records, and classified as whether occurring within 12 months of diagnosis [10]. Procedure codes for chemotherapy delivery were obtained from admission records, MBS items, and also PBS items classified using WHO Anatomical Therapeutic Clinical Classification System (ATC) codes, where identified as occurring within 12 months of diagnosis or not [16]. Specific codes for targeted and hormone therapy were identified in PBS data and dates of supply were used to indicate administration beginning within 12 months of diagnosis.

Clinical care and survival were analysed by level of socioeconomic disadvantage (Quintiles 1–2 versus 3–5) and of residential remoteness (remote/very remote versus major city/inner regional/outer regional) [18, 19].

### Statistical analyses

Descriptive statistics were calculated for all women, including those residing in border local health districts, across study variables, collapsing rows or columns as needed to avoid cell sizes < 5. Descriptive statistics for treatment variables were calculated for NSW residents who resided outside of local health districts adjacent to the NSW border. Crude associations were calculated by logistic regression, and then with adjustment for sociodemographic (i.e., age, country of birth, diagnostic period, histology type, extent of disease, and residential IRSD and remoteness ARIA) and clinical factors. Clinical exposures examined were any treatment, any surgery, type of surgery (breast conserving or mastectomy), radiotherapy following breast conserving surgery, and reconstruction following mastectomy. Penalized logistic regression was used to model associations between study variables and remoteness to avoid issues of sparse data and separation [21, 22]. Crude and adjusted logistic regressions involving treatment variables (including penalized logistic regression) excluded those women in border local health districts (*n* = 6447).

Disease-specific survivals at 1 year, 2 year, 3 year and 5 years following diagnosis were calculated using Kaplan Meier product-limit estimates [23] for all women including those living in border local health districts. These estimates have been shown to be good proxies for relative survival in Australia where causes of death are assigned by registry staff based on cancer notifications, hospital reporting and death records [24]. This is important, given the uncertain accuracy of causes of death recorded on death registrations in many international studies [25]. Disease-specific survival was chosen because life tables were not available for many comparison groups to use net survival [23].

In this study, survival times were measured from diagnosis until death or censoring on December 31, 2015, whichever occurred first. Corresponding measures of breast cancer survival were obtained using competing risk regression (competing deaths being those from causes other than breast cancer) [26]. Predictors included age at diagnosis, residential remoteness, socioeconomic status, country of birth, diagnostic period, histology type and stage (extent of disease). The proportional hazards assumption was tested, and for variables where it was found not to be met, interaction terms with natural log time (years) were included.

All analyses were undertaken using Stata 16.0 [23].

## Results

### Descriptive characteristics

**Fig. 1:**
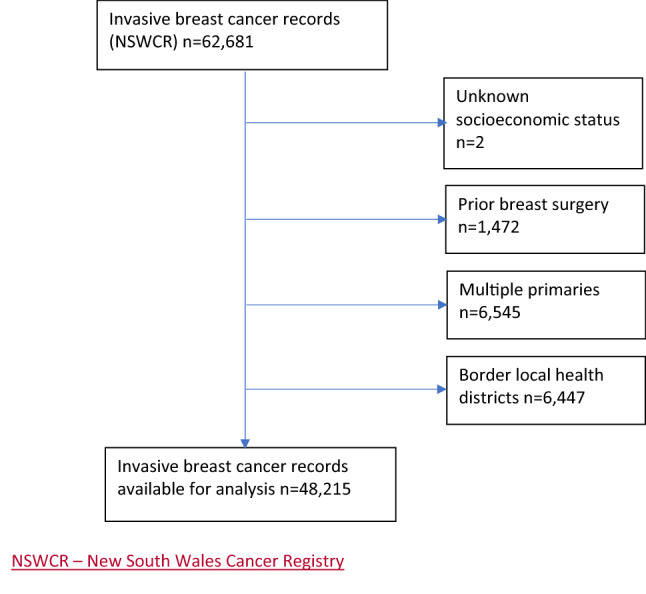
Application of inclusion/exclusion criteria for analysis

There were 62,681 women initially included in the linked dataset (Fig. 1). After excluding those of unknown socioeconomic status (*n* = 2), those having prior breast surgery (*n* = 1472), those living in a border local health district (*n* = 6,447) and those with multiple primaries (*n* = 6545), 48,215 were available for analysis of treatment variables, and 54,662 for survival analyses.

Approximately half (51%) these women were aged from 50 to 69 years (Table 1). Over 70% lived in major city areas and 43% in areas of socioeconomic advantage (IRSD Q4/5). The proportion living in areas of greatest socioeconomic disadvantage (Q1) comprised 17%. Approximately two thirds (67%) were born in Australia, 11% in other mainly English-speaking countries and 23% in mainly non-English speaking countries. More than half (52%) of those with known stage were diagnosed with local disease and approximately 6% with distant disease. The percentage treated for breast cancer within 12 months of diagnosis was 92% for breast surgery (53% for breast conserving surgery and 39% for mastectomy), 60% for radiotherapy, 44% for chemotherapy, 67% for hormone therapy, and 8% for immunotherapy. Five percent had breast reconstruction (i.e., 14% of those having a mastectomy).

**Table 1:** Descriptive statistics for women with invasive breast cancer, NSW 2002–2015

Variable	N (%)
All	54,662 (100)
*Age group (years)*	
< 40	3,212 (5.9)
40–49	10,177 (18.6)
50–59	14,194 (26.0)
60–69	13,855 (25.4)
70–79	8,255 (15.1)
80 +	4,969 (9.1)
*Remoteness*	
Metropolitan	38,670 (70.7)
Inner regional	12,148 (22.2)
Outer regional	3,596 (6.6)
Remote & very remote	248 (< 1)
*Socioeconomic status (quintiles)*	
1 (Most disadvantaged)	9,304 (17.0)
2	11,221 (20.5)
3	10,702 (19.6)
4	10,665 (19.5)
5 (Least disadvantaged)	12,770 (23.4)
*Country of Birth*	
Australia	36,517 (66.8)
Other English-speaking countries	5,741 (10.5)
Non-English-speaking countries	12,404 (22.7)
*Diagnosis Year*	
2002–2007	19,805 (36.2)
2008–2015	34,857 (63.8)
*Histology*	
Ductal	40,596 (74.3)
Lobular	5,778 (10.6)
Other	8,288 (15.2)
*Extent of disease*	
Local	28,158 (51.5)
Regional	20,523 (37.6)
Distant	3,173 (5.8)
Unknown	2,808 (5.1)
*In women residing outside of border local health districts (n* = *48,215)*	
*Surgery within 12 months*	
None	3,845 (8.0)
Breast conserving	25,517 (52.9)
Mastectomy	18,853 (39.1)
*Reconstruction following mastectomy (n* = *18,853)*	
Immediate	1,043 (5.5)
Within 12 months	852 (4.5)
Within 5 years	681 (3.6)
None	16,277 (86.3)
*Radiotherapy within 12 months*	
Yes	28,869 (59.9)
No	19,346 (40.1)
*Chemotherapy within 12 months*	
Yes	21,272 (44.1)
No	26,943 (55.9)
*Hormone therapy within 12 months*	
Yes	32,116 (66.6)
No	16,099 (33.4)
*Immunotherapy within 12 months*	
Yes	3,843 (8.0)
No	44,372 (92.0)

### Unadjusted comparisons by socioeconomic status and remoteness

#### Socioeconomic status

Women aged 50–79 years at diagnosis had 11–29% higher odds of living in areas of greater socioeconomic disadvantage than those aged < 40 years (Table 2). Living in inner regional, very remote and, more so, outer regional areas was strongly associated with socioeconomic disadvantage (*ORunadj* 39.4 [95% CI 32.9, 47.1] for outer regional). Women who were born in other mainly English-speaking countries had 27% lower odds than women born in Australia of living in areas of greater socioeconomic disadvantage (*ORunadj* 0.73 [95% CI 0.68, 0.78]), whereas those born in other mainly non-English-speaking countries had 16% higher odds of living in areas of socioeconomic disadvantage.

**Table 2:** Unadjusted logistic regression on socioeconomic disadvantage (Quintile 1 and Quintile 2) and remoteness (remote and very remote), respectively

Independent variables	Odds Ratio [95% CI] (n = 48,215)	
	Socioeconomic disadvantage (Q1&Q2)	Remote & very remote^a^
*Age group (years)*		
< 40	1.0 (reference)	1.0 (reference)
40–49	1.03 [0.94, 1.13]	1.57 [0.75, 3.28]
50–59	1.11 [1.02, 1.21]	1.66 [0.71, 3.40]
60–69	1.24 [1.13, 1.35]	1.44 [0.69, 2.97]
70–79	1.29 [1.18, 1.41]	1.70 [0.81, 3.61]
80 +	1.08 [0.97, 1.19]	1.07 [0.45, 2.53]
*Remoteness*		
Metropolitan	1.0 (reference)	
Inner regional	3.59 [3.41, 3.77]	
Outer regional	39.36 [32.90, 47.09]	
Remote & very remote	141.27 [52.50, 380.14]	
*Socioeconomic status (quintiles)*		
1 (Most disadvantaged)		1.0 (reference)
2		0.23 [0.17, 0.32]
3		< 0.01 [< 0.01, 0.04]
4		0.02 [< 0.01, 0.05]
5 (Least disadvantaged)		< 0.01 [< 0.01, 0.03]
*Country of Birth*		
Australia	1.0 (reference)	1.0 (reference)
Other English-speaking countries	0.73 [0.68, 0.78]	0.30 [0.16, 0.58]
Non-English-speaking countries	1.16 [1.11, 1.21]	0.15 [0.08, 0.27]
*Diagnosis Year*		
2002–2007	1.0 (reference)	1.0 (reference)
2008–2015	0.96 [0.92, 0.99]	0.92 [0.70, 1.22]
*Histology*		
Ductal	1.0 (reference)	1.0 (reference)
Lobular	0.89 [0.84, 0.95]	0.72 [0.43, 1.19]
Other	0.93 [0.88, 0.98]	0.95 [0.65, 1.39]
*Extent of disease*		
Local	1.0 (reference)	1.0 (reference)
Regional	1.00 [0.96, 1.05]	0.91 [0.68, 1.23]
Distant	1.31 [1.21, 1.42]	1.14 [0.66, 1.97]
Unknown	1.02 [0.93, 1.12]	1.0 [0.53, 1.89]
*Any treatment within 12 months*		
No	1.0 (reference)	1.0 (reference)
Yes	0.95 [0.82, 1.09]	0.64 [0.28, 1.51]
*Any surgery within 12 months*		
No	1.0 (reference)	1.0 (reference)
Yes	0.86 [0.80, 0.92]	0.79 [0.50, 1.25]
*Surgery within 12 months*		
None	1.0 (reference)	1.0 (reference)
Breast conserving	0.83 [0.77, 0.89]	0.75 [0.47, 1.21]
Mastectomy	0.90 [0.84, 0.97]	0.85 [0.52, 1.38]
*Reconstruction*^b^		
Immediate	0.38 [0.33, 0.45]	0.10 [0.01, 1.55]
Within 12 months	0.35 [0.29, 0.42]	0.11 [0.01, 1.84]
Within 5 years	0.53 [0.44, 0.63]	0.72 [0.20, 2.53]
None	1.0 (reference)	1.0 (reference)
*Radiotherapy after BCS*		
No	1.0 (reference)	1.0 (reference)
Yes	0.85 [0.80, 0.91]	0.73 [0.47, 1.15]
*Chemotherapy within 12 months*		
No	1.0 (reference)	1.0 (reference)
Yes	0.99 [0.95, 1.03]	1.12 [0.85, 1.47]
*Hormone therapy within 12 months*		
None	1.0 (reference)	1.0 (reference)
Yes	0.94 [0.91, 0.98]	1.04 [0.78, 1.40]
*Immunotherapy within 12 months*		
None	1.0 (reference)	1.0 (reference)
Yes	1.01 [0.94, 1.08]	1.06 [0.65, 1.73]

*CI* confidence interval, *Q1 & Q2* quintile 1 & quintile 2), *BCS* breast conserving surgery

^a^Penalized logistic regression

^b^reconstruction after total mastectomy

Women with cancers of non-ductal histology type were less likely to live in areas of greater socioeconomic disadvantage (36% and 38%, respectively; OR*unadj* 0.89 [95% CI 0.84, 0.95]). Being diagnosed with distant disease was also more likely to occur in women living in socioeconomically disadvantaged areas (*ORunadj* 1.31 [95% CI 1.21, 1.42]). Women undergoing any surgery (breast conserving or mastectomy) were less likely to live in areas of greater socioeconomic disadvantage, as were those receiving radiotherapy, hormone therapy, and breast reconstruction after mastectomy (Table 2).

### Residential remoteness

Women living in more advantaged areas were less likely to live in more remote areas (Table 2). Those born outside of Australia were less likely to live in more remote areas compared with women born in Australia, especially if born in mainly non-English speaking countries with OR*unadj* 0.30 [95% CI 0.16, 0.58] and (OR*unadj* 0.15 [95% CI 0.08, 0.27]) respectively.

Although not reaching statistical significance, women undergoing reconstruction after mastectomy were less likely to reside in more remote areas (Table 2). Similarly, any treatment, or treatment by radiotherapy after breast conserving surgery was less common for residents of more remote areas at OR*unadj* 0.64 [0.28, 1.51and OR*unadj* 0.73 [0.47, 1.15], respectively (Table 2).

### Adjusted comparisons by socioeconomic status and remoteness

#### Socioeconomic status

Five multivariate logistic regression models were used to predict the odds of socioeconomic disadvantage. All included age at diagnosis, residential remoteness, country of birth, histology type, stage, and diagnostic year, plus selected treatments, i.e.: any treatment (Model 1), and in addition to radiotherapy, chemotherapy, hormone therapy and immunotherapy, any surgery (Model 2), surgery type (Model 3), all variables limited to women having breast conserving surgery (Model 4); and breast reconstruction in women having a mastectomy (Model 5).

From Table 3, it is evident that odds of residing in a socioeconomically disadvantaged area were related to:

- Age at diagnosis – compared with < 40 years, (a) elevated odds for 70–79 years in Models 1–4 and 60–69 years in Models 2–4; and (b) lower odds in Model 5 for ages 40–59 and 80 + years.
- Residential remoteness – compared with metropolitan areas, progressively higher odds for more remote areas in all Models.
- Country of birth – compared with Australia, elevated odds for mainly non-English speaking countries in all Models, and lower odds for mainly English-speaking countries in all Models.
- Diagnosis year – compared with 2002–2007, lower odds for 2008–2015 in Models 1–3.
- Histology type – compared with ductal, lower odds for lobular in Models 1–3 and 5.
- Stage (extent of disease) – compared with local disease, elevated odds for distant in all Models and for regional in Models 1–3.
- Breast reconstruction (mastectomy cases) – compared with no reconstruction, lower odds for immediate reconstruction and delayed reconstruction (12 month) in Model 5.
- Radiotherapy – lower odds for Models 2 and 4.
- Chemotherapy – higher odds for Model 4.
- Hormone therapy – lower odds for models 2–4.

**Table 3:** Association of sociodemographic, clinical and treatment factors with living in socioeconomically disadvantaged (IRSD Quntiles 1-2) areas in NSW

Independent variables	Odds Ratio [95% CI] Socioeconomic disadvantage				
	Model 1 (any treatment)	Model 2 (any surgery)	Model 3 (surgery type)	Model 4 (BCS cases)	Model 5 (Mastectomy cases)^a^
*Age group (years)*					
< 40	1.0 (reference)	1.0 (reference)	1.0 (reference)	1.0 (reference)	1.0 (reference)
40–49	0.96 [0.88, 1.06]	0.97 [0.88, 1.07]	0.97 [0.88, 1.07]	1.00 [0.87, 1.16]	0.86 [0.75, 0.98]
50–59	1.01 [0.92, 1.11]	1.02 [0.93, 1.12]	1.03 [0.94, 1.13]	1.05 [0.91, 1.21]	0.85 [0.74, 0.98]
60–69	1.09 [0.99, 1.19]	1.11 [1.01, 1.22]	1.11 [1.01, 1.22]	1.09 [0.94, 1.26]	0.93 [0.81, 1.08]
70–79	1.15 [1.04, 1.27]	1.17 [1.06, 1.29]	1.17 [1.06, 1.30]	1.14 [0.98, 1.33]	0.93 [0.80, 1.08]
80 +	1.00 [0.89, 1.11]	1.00 [0.89, 1.12]	1.00 [0.89, 1.12]	1.00 [0.83, 1.20]	0.73 [0.61, 0.87]
*Remoteness*					
Metropolitan	1.0 (reference)	1.0 (reference)	1.0 (reference)	1.0 (reference)	1.0 (reference)
Inner regional	4.22 [4.00, 4.44]	4.21 [4.00, 4.44]	4.21 [4.00, 4.43]	4.05 [3.78, 4.35]	4.56 [4.20, 4.95]
Outer regional	47.33 [39.52, 56.68]	47.11 [39.4, 56.42]	47.10 [39.33, 56.40]	43.82 [34.30, 55.97]	48.99 [36.79, 65.24]
Remote/very remote	168.29 [62.52, 453.01]	167 [62.18, 450.56]	167.37 [62.17, 450.53]	112.35 [35.59, 54.66]	522.51 [32.41, 8423.96]
*Country of Birth*					
Australia	1.0 (reference)	1.0 (reference)	1.0 (reference)	1.0 (reference)	1.0 (reference)
Other English-speaking countries	0.87 [0.81, 0.93]	0.87 [0.81, 0.93]	0.87 [0.81, 0.93]	0.87 [0.79, 0.96]	0.86 [0.77, 0.97]
Non-English-speaking countries	1.89 [1.80, 1.98]	1.88 [1.79, 1.97]	1.88 [1.79, 1.97]	1.90 [1.77, 2.03]	1.83 [1.69, 1.97]
*Diagnosis Year*					
2002–2007	1.0 (reference)	1.0 (reference)	1.0 (reference)	1.0 (reference)	1.0 (reference)
2008–2015	0.94 [0.91, 0.98]	0.95 [0.91, 0.99]	0.95 [0.91, 0.99]	0.96 [0.90, 1.02]	0.99 [0.93, 1.07]
*Histology*					
Ductal	1.0 (reference)	1.0 (reference)	1.0 (reference)	1.0 (reference)	1.0 (reference)
Lobular	0.89 [0.83, 0.95]	0.90 [0.84, 0.96]	0.90 [0.84, 0.96]	0.92 [0.83, 1.02]	0.90 [0.82, 1.0]
Other	0.96 [0.91, 1.02]	0.95 [0.90, 1.01]	0.95 [0.90, 1.01]	0.96 [0.89, 1.05]	0.97 [0.88, 1.06]
*Extent of disease*					
Local	1.0 (reference)	1.0 (reference)	1.0 (reference)	1.0 (reference)	1.0 (reference)
Regional	1.07 [1.02, 1.12]	1.06 [1.01, 1.11]	1.06 [1.01, 1.11]	1.04 [0.97, 1.11]	1.05 [0.97, 1.13]
Distant	1.41 [1.29, 1.53]	1.34 [1.22, 1.47]	1.33 [1.21, 1.47]	1.34 [1.12, 1.60]	1.25 [1.07, 1.47]
Unknown	1.02 [0.92, 1.12]	0.96 [0.86, 1.06]	0.96 [0.86, 1.06]	0.97 [0.81, 1.17]	1.03 [0.84, 1.27]
*Any treatment within 12 months*					
No	1.0 (reference)				
Yes	1.07 [0.91, 1.26]				
*Any surgery within 12 months*					
No		1.0 (reference)			
Yes		0.93 [0.85, 1.02]			
*Surgery within 12 months*					
None			1.0 (reference)		
Breast conserving			0.92 [0.84, 1.01]		
Mastectomy			0.94 [0.86, 1.03]		
*Reconstruction*					
None					1.0 (reference)
Immediate					0.49 [0.41, 0.58]
12 month					0.42 [0.34, 0.51]
5 year					0.61 [0.51, 0.75]
*Radiotherapy within 12 months*					
No		1.0 (reference)	1.0 (reference)	1.0 (reference)	1.0 (reference)
Yes		0.95 [0.91, 0.99]	0.95 [0.91, 1.00]	0.90 [0.83, 0.97]	1.01 [0.93, 1.09]
*Chemotherapy within 12 months*					
No		1.0 (reference)	1.0 (reference)	1.0 (reference)	1.0 (reference)
Yes		1.04 [0.99, 1.09]	1.04 [0.98, 1.09]	1.09 [1.01, 1.16]	0.94 [0.87, 1.02]
*Hormone therapy within 12 months*					
No		1.0 (reference)	1.0 (reference)	1.0 (reference)	1.0 (reference)
Yes		0.95 [0.91, 0.99]	0.95 [0.91, 0.99]	0.93 [0.88, 0.99]	0.95 [0.88, 1.02]
*Immunotherapy within 12 months*					
No		1.0 (reference)	1.0 (reference)	1.0 (reference)	1.0 (reference)
Yes		1.02 [0.94, 1.10]	1.01 [0.94, 1.10]	1.02 [0.91, 1.15]	1.01 [0.90, 1.13]

*NSW* New South Wales, *IRSD* Index of Relative Socioeconomic Disadvantage, *BCS* breast conserving surgery, *CI* confidence interval

^a^Penalized logistic regression

### Residential remoteness

From Table 4 it is evident that odds of residing in more remote areas were related to:

- Age at diagnosis – compared with < 40 years, elevated odds for older ages in Models 1 and 4, and for 40–79 years in Models 2 and 3.
- Socioeconomic disadvantage – compared with most disadvantaged, progressively lower odds for lesser disadvantaged areas.
- Country of birth – compared with Australia, lower odds for mainly English-speaking countries and (more so) for mainly non-English countries in all Models.
- Stage (extent of disease) – compared with local, lower odds for regional and (more so) distant stage in Models 1–4.
- Any treatment – lower odds in Model 1.
- Breast reconstruction (mastectomy cases) – compared with no reconstruction, lower odds for immediate reconstruction in Model 5.
- Radiotherapy – lower odds for all Models.

**Table 4:** Association of sociodemographic, clinical and treatment factors with living in remote and very remote NSW

Independent variables		Odds Ratio [95% CI] Remoteness				
		Model 1 (any treatment)	Model 2 (any surgery)	Model 3 (surgery type)	Model 4 (in BCS cases)	Model 5 (Mastectomy cases)
*Age group (years)*						
< 40		1.0 (reference)	1.0 (reference)	1.0 (reference)	1.0 (reference)	1.0 (reference)
40–49		1.61 [0.76, 3.40]	1.63 [0.77, 3.43]	1.63 [0.77, 3.45]	2.25 [0.60, 8.40]	1.24 [0.47, 3.30]
50–59		1.56 [0.76, 3.24]	1.62 [0.78, 3.36]	1.62 [0.78, 3.37]	1.72 [0.46, 6.35]	1.43 [0.55, 3.70]
60–69		1.32 [0.63, 2.76]	1.38 [0.65, 2.91]	1.38 [0.65, 2.92]	1.61 [0.43, 6.05]	1.05 [0.39, 2.81]
70–79		1.50 [0.70, 3.21]	1.53 [0.70, 3.34]	1.53 [0.70, 3.36]	1.91 [0.49, 7.54]	0.91 [0.31, 2.67]
80 +		0.99 [0.41, 2.38]	0.96 [0.38, 2.39]	0.96 [0.38, 2.41]	2.09 [0.46, 9.38]	0.81 [0.22, 2.92]
*Socioeconomic status (quintiles)*						
1 (Most disadvantaged)		1.0 (reference)	1.0 (reference)	1.0 (reference)	1.0 (reference)	1.0 (reference)
2		0.17 [0.12, 0.24]	0.17 [0.12, 0.24]	0.17 [0.12, 0.24]	0.17 [0.12, 0.24]	0.21 [0.13, 0.34]
3		< 0.01 [< 0.01, 0.03]	< 0.01 [< 0.01, 0.03]	< 0.01 [< 0.01, 0.03]	< 0.01 [< 0.01, 0.03]	< 0.01 [< 0.01, 0.08]
4		0.02 [0.01, 0.05]	0.02 [0.01, 0.05]	0.02 [0.01, 0.05]	0.02 [0.01, 0.05]	< 0.01 [< 0.01, 0.09]
5 (Least disadvantaged)		< 0.01 [< 0.01, 0.03]	< 0.01 [< 0.01, 0.03]	< 0.01 [< 0.01, 0.03]	< 0.01 [< 0.01, 0.03]	< 0.01 [< 0.01, 0.08]
*Country of Birth*						
Australia		1.0 (reference)	1.0 (reference)	1.0 (reference)	1.0 (reference)	1.0 (reference)
Other English-speaking countries		0.39 [0.20, 0.74]	0.39 [0.20, 0.75]	0.39 [0.20, 0.75]	0.72 [0.35, 1.46]	0.17 [0.03, 0.86]
Non-English-speaking countries		0.08 [0.04, 0.16]	0.08 [0.05, 0.16]	0.08 [0.05, 0.16]	0.10 [0.04, 0.23]	0.11 [0.05, 0.26]
*Diagnosis Year*						
2002–2007		1.0 (reference)	1.0 (reference)	1.0 (reference)	1.0 (reference)	1.0 (reference)
2008–2015		1.00 [0.75, 1.33]	1.02 [0.76, 1.37]	1.02 [0.76, 1.36]	1.23 [0.80, 1.89]	0.95 [0.60, 1.48]
*Histology*						
Ductal		1.0 (reference)	1.0 (reference)	1.0 (reference)	1.0 (reference)	1.0 (reference)
Lobular		0.79 [0.47, 1.32]	0.80 [0.46, 1.31]	0.78 [0.46, 1.31]	1.15 [0.58, 2.30]	0.64 [0.30, 1.39]
Other		1.01 [0.68, 1.49]	1.03 [0.70, 1.53]	1.03 [0.70, 1.53]	0.93 [0.51, 1.67]	0.89 [0.46, 1.72]
*Extent of disease*						
Local		1.0 (reference)	1.0 (reference)	1.0 (reference)	1.0 (reference)	1.0 (reference)
Regional		0.95 [0.70, 1.28]	0.90 [0.65, 1.25]	0.90 [0.65, 1.25]	1.17 [0.74, 1.84]	0.68 [0.40, 1.13]
Distant		0.88 [0.50, 1.56]	0.77 [0.41, 1.45]	0.77 [0.41, 1.45]	0.79 [0.22, 2.89]	0.85 [0.33, 2.19]
Unknown		1.04 [0.53, 2.01]	0.94 [0.47, 1.90]	0.94 [0.47, 1.90]	1.71 [0.64, 4.56]	1.32 [0.43, 4.08]
*Any treatment within 12 months*						
No		1.0 (reference)				
Yes		0.49 [0.19, 1.22]				
*Any surgery within 12 months*						
No			1.0 (reference)			
Yes			0.76 [0.44, 1.33]			
*Surgery within 12 months*						
None				1.0 (reference)		
Breast conserving				0.75 [0.42, 1.36]		
Mastectomy				0.77 [0.43, 1.37]		
*Reconstruction*						
None						1.0 (reference)
Immediate						0.17 [0.01, 2.82]
12 month						0.18 [0.01, 3.02]
5 year						0.88 [0.24, 3.22]
*Radiotherapy within 12 months*						
*No*			1.0 (reference)	1.0 (reference)	1.0 (reference)	1.0 (reference)
Yes			0.75 [0.56, 1.01]	0.76 [0.55, 1.07]	0.77 [0.47, 1.28]	0.74 [0.43, 1.27]
*Chemotherapy within 12 months*						
No			1.0 (reference)	1.0 (reference)	1.0 (reference)	1.0 (reference)
Yes			1.17 [0.83, 1.65]	1.16 [0.82, 1.65]	1.12 [0.68, 1.85]	1.22 [0.70, 2.13]
*Hormone therapy within 12* *months*						
No			1.0 (reference)	1.0 (reference)	1.0 (reference)	1.0 (reference)
Yes			1.13 [0.84, 1.53]	1.13 [0.84, 1.53]	0.96 [0.63, 1.46]	1.36 [0.84, 2.21]
*Immunotherapy within 12 months*						
No			1.0 (reference)	1.0 (reference)	1.0 (reference)	1.0 (reference)
Yes			0.99 [0.48, 1.69]	0.99 [0.58, 1.69]	1.20 [0.58, 2.52]	0.82 [0.37, 1.84]

^a^Penalized logistic regression of cohort without borders LHDs

*NSW* New South Wales, *BCS* breast conserving surgery, *CI* confidence interval

### Disease-specific survival

#### Unadjusted comparisons

Differences in survival were very small by area of disadvantage albeit achieving statistical significance (Table 5). The 5-year survival was 89% for the more disadvantaged areas compared with 90% for the less disadvantaged. Differences were smaller again and not statistically significant by residential remoteness, with a 5-year survival of 90% presenting (Table 5).

**Table 5:** Percentage Kaplan–Meier survival and 95% confidence intervals

Years since diagnosis	Socioeconomic status		Remoteness	
	Q1-Q2	Q3-Q5	M/IR/OR	R/VR
1	97.8 [97.5, 98.0]	98.2 [98.1, 98.4]	98.1 [97.9, 98.2]	98.5 [95.4, 99.5]
2	95.5 [95.1, 95.8]	96.1 [95.9, 96.3]	95.9 [95.7, 96.1]	97.4 [93.9, 98.9]
3	93.0 [92.5, 93.4]	94.1 [93.7, 94.3]	93.6 [93.4, 93.9]	93.7 [88.9, 96.5]
5	88.6 [88.1, 89.2]	90.4 [90.0, 90.8]	89.8 [89.5, 90.1]	90.3 [84.5, 94.0]
10	82.5 [81.7, 83.3]	85.0 [84.5, 85.6]	84.2 [83.7, 84.6]	83.4 [75.6, 88.9]

Q1-Q2 = quintile 1-quintile 2; Q3-Q5 = quintile 3-quintile 5; M/IR/OR = metropolitan/inner regional/outer regional; R/VR = remote/very remote

#### Adjusted comparisons

Multivariate competing risk regression showed an elevated risk of breast cancer death in women aged 70 + years compared with < 40 years, after adjusting for other characteristics in the model (Table 6). The more advanced the stage, the higher was the risk of death. Women born in mainly non-English speaking countries were less likely to have a recorded breast cancer death than the Australian born. There were also differences by histology type where, compared with ductal lesions, the risk of death was lower for women with lobular but higher for those with other histology types. A lower risk applied to 2008–2015 than in 2002–2007 diagnoses with *sHRadjusted* = 0.74 [0.70, 0.79]. Differences in risk of breast cancer death were not evident by residential remoteness nor by socioeconomic disadvantage for four of the five quintiles. Compared with the most disadvantaged, however, the least disadvantaged had a lower risk of breast cancer death with sHR*adjusted* = 0.86 [0.79, 0.94].

**Table 6:** Sociodemographic and clinical predictors of breast cancer mortality in NSW breast cancer cases (n = 54,662), 2002–2015

	Subhazard ratios [95% CI]
*Age group (years)*	
< 40	1.0
40–49	0.80 [0.66, 0.99]
50–59	1.03 [0.85, 1.24]
60–69	1.18 [0.97, 1.42]
70–79	2.16 [1.79, 2.60]
80 +	3.22 [2.67, 3.90]
*Remoteness*	
Metropolitan	1.0
Inner regional	0.98 [0.92, 1.05]
Outer regional	1.08 [0.96, 1.20]
Remote & very remote	0.95 [0.67, 1.34]
*Socioeconomic status (quintiles)*	
1 (Most disadvantaged)	1.0
2	1.04 [0.96, 1.13]
3	0.94 [0.86, 1.03]
4	0.95 [0.87, 1.04]
5 (Least disadvantaged)	0.86 [0.79, 0.94]
*Country of Birth*	
Australia	1.0
Other English-speaking countries	0.97 [0.86, 1.08]
Non-English-speaking countries	0.80 [0.73, 0.87]
*Diagnosis year*	
2002–2007	1.0
2008–2015	0.74 [0.70, 0.79]
*Histology*	
Ductal	1.0
Lobular	0.83 [0.73, 0.94]
Other	1.14 [1.05, 1.24]
*Extent of disease*	
Local	1.0
Regional	5.19 [4.54, 5.94]
Distant	36.79 [32.14, 42.10]
Unknown	7.02 [5.95, 8.29]

Model included interaction terms with time for age group, histology, extent of disease and country of birth;

*NSW * New South Wales, *CI*  confidence interval

1. Metadata Online Registry (2012) AIHW. https://meteor.aihw.gov.au/content/index.phtml/itemId/480010

## Discussion

The present results are reassuring in indicating a high five-year disease-specific survival from breast cancer at the high end of the international scale at 90% in New South Wales (NSW), and with evidence of an upward trend. These is little indication of differences in crude survival by residential remoteness despite large distances that many women have to travel for diagnosis and treatment and only a marginal difference by socioeconomic status. Equivalent adjusted survival applied in four of the five socioeconomic quintiles, but with the highest socioeconomic quintile showing higher survival.

Sub-hazard ratios indicated higher risks of death from breast cancer in older cases aged 70 years or more, and predictably, with more advanced stage (extent of disease), which is consistent with previous studies [2, 4]. While higher survival was found for women born in non-English speaking countries than for the Australian-born, confirmatory evidence is needed, given the potential for bias (e.g., from missed recording of deaths for women returning to their birth country with terminal disease) [10].

Adjusted analyses indicated more advanced stage in lower socioeconomic areas, with women from these areas presenting less evidence of breast reconstruction following mastectomy. Also, following breast conserving surgery, women from lower socioeconomic areas were less likely to have radiotherapy, and hormone therapy, but more likely to have chemotherapy.

Remoteness of residence was also associated in adjusted analyses with less radiotherapy and less use of immediate breast reconstruction. The lesser use of radiotherapy applied in all women, including those having breast conserving surgery. Contrary to earlier studies, however, remoteness of residence was not associated with lower survival or more advanced stage [2, 27].

Common to both the more socioeconomically disadvantaged and more remote areas was the lower exposure to radiotherapy and breast reconstruction. Adjuvant radiotherapy with breast conserving surgery is equivalent to mastectomy for overall, disease-free survival, and distant-metastasis free survival [28, 29]. The lower likelihood of receiving adjuvant radiotherapy in breast conserving surgery cases with lower socioeconomic status may have contributed to the higher breast cancer survival seen in the least disadvantaged quintile.

Difficulties accessing radiotherapy services have been documented previously [30]. For example, some women from rural areas, particularly those of older age, may decline treatment recommendations due to the inconvenience of travelling, anticipated extended stays away from home, and limited social support available in their areas [31–33]. Greater use of hypofractionation protocols is occurring, however, which may lead to increased uptake of radiotherapy and concomitant reductions in mastectomies in clinical situations where otherwise breast conserving surgery/radiotherapy is an evidence-based option, including in women of lower socioeconomic status who may face more financial pressures through loss of work and family costs associated with attending radiotherapy [32, 33].

Considerable effort and investment are being made to address access issues [34]. Cancer care centres in NSW are now readily accessible (within 100 km) for 95% of the population [35]. The extent to which remaining access issues are deterring radiotherapy requires further research.

Reconstruction is preferred by many women to minimise impact on body image following breast cancer surgery [36, 37]. However, the proportion of women having this treatment following a mastectomy is low in NSW (and in Australia overall). Private health insurance and younger age are strong predictors of breast reconstruction [38]. This study found immediate reconstruction to be negatively associated with residential remoteness and strongly associated with higher socioeconomic status.

Universal Health Insurance in Australia (Medicare) covers breast reconstruction costs in the private sector. Nonetheless, waiting times can be an issue in the public sector, such that public patients may be less likely than those in the private sector to obtain these services [39]. Women with comorbidity are less likely to have reconstruction which may contribute to the association of lower reconstruction rates with socioeconomic status, as seen in the present study [40, 41].

Further research is needed into cultural differences by socioeconomic status and remoteness that may also affect uptake of breast reconstruction following mastectomy. Meanwhile, promoting greater discussion between clinicians and patients regarding the option of breast reconstruction, and also of means of improving the acceptability of the procedure, may serve to minimise variation across sociodemographic groups [42, 43]. As some women living remotely are more likely to have mastectomy due to barriers to receiving adjuvant therapy, it is important that opportunities for breast reconstruction are readily available to them.

Recent data show a lower cancer survival for people living in more socioeconomically disadvantaged areas [10]. We found breast cancer survival and associated factors largely align with other reports with lower breast cancer mortality occurring in those living in areas of least socioeconomic disadvantage at diagnosis [44, 45]. The present data show little difference in survival by remoteness of residence, however, which is consistent with earlier research [46].

The strengths of this study are the-whole-of population approach to breast cancer analysis in NSW. Understanding real-world treatment patterns is important to interpreting the progressive increases in survival. Data linkage provides the opportunity to investigate treatment patterns at an all-of-health system level. Monitoring of differences in treatment, particularly for priority populations, are important to identify areas of unmet need for policy development.

Limitations of this study include the absence of biomarker data to enable assessment of the appropriate use of immunotherapies and hormone therapy. Other investigators are analysing Australian data on the patterns of use and outcomes for HER2 positive women, showing for example that women receiving trastuzumab are less likely to complete treatment if living in areas of socioeconomic disadvantage, or in remote areas [47]. The analysis of associations between treatment and remoteness were limited by the smaller population of women living in remote and very remote areas, which reduced the statistical power available to reveal differences.

## Conclusions

Survival from breast cancer is high in NSW by world standards and increased further between 2002–2007 and 2008–2015. Little evidence of survival differences presents in adjusted analyses by remoteness but there is evidence that residents of areas in highest socioeconomic quintile have higher survival. Policy makers should continue their focus on initiatives to improve survival further by addressing barriers to cancer care, particularly as related to use of radiotherapy following breast conserving surgery and breast reconstruction following mastectomy, whether due to socioeconomic or geographic barriers.

## Declarations

## Data Availability

The data that support the findings of this study are available from the Cancer Institute NSW and Australian Institute of Health and Welfare, but restrictions apply to the availability of these data, which were used under license for the current study, and so are not publicly available. However, data are available from the authors upon reasonable request and with permission of the NSW Cancer Institute and Australian Institute of Health and Welfare.

## Abbreviations

APDC: Admitted Patient Data Collection
ARIA: Accessibility Remoteness Index of Australia
BCS: Breast conserving surgery
CI: Confidence interval
HER2: Human Epidermal Growth Factor receptor 2
ICD-O-3: International Classification of Diseases for Oncology, third version
IRSD: Index of Relative Socioeconomic Disadvantage
LHDs: Local health districts
MBS: Medicare Benefits Schedule
PBS: Pharmaceutical Benefits Schedule
NSW: New South Wales
NSWCR: New South Wales Cancer Registry
SURE: Secure Unified Research Environment
